# Integrating Multifunctional Hydrogen-Bonded Organic Frameworks into Intelligent Packaging: Mechanisms, Design and Challenges

**DOI:** 10.3390/ma19061254

**Published:** 2026-03-22

**Authors:** Yabo Fu, Yubing Zhang, Congyao Wang, Jingmei Guan, Jiazi Shi, Hui Liu, Bo Lu

**Affiliations:** 1 Beijing Key Laboratory of Printing Packaging Materials and Technology, Beijing 102600, China; fuyabo@126.com (Y.F.); 13582326500@163.com (Y.Z.); 15081122858@163.com (C.W.); gjm20201574@163.com (J.G.); shijiazi@bigc.edu.cn (J.S.); liuhui@bigc.edu.cn (H.L.); 2 School of Printing and Packaging Engineering, Beijing Institute of Graphic Communication, Beijing 102600, China

**Keywords:** smart packaging, hydrogen-bonded organic frameworks, porous materials, mechanisms, regulating

## Abstract

The transition from passive containment to active, responsive management is defining the next generation of intelligent packaging. This evolution creates a critical demand for materials that can be precisely engineered to monitor, regulate, and protect. Hydrogen-bonded organic frameworks (HOFs) have emerged as a uniquely versatile platform in this regard, owing to their synthetically tunable porosity, inherent biocompatibility, and exceptional solution processability derived from reversible supramolecular assembly. This review moves beyond cataloging applications to dissect the fundamental mechanisms by which HOFs enable smart packaging functions, including the following: (i) selective gas capture and atmosphere tailoring via molecular recognition within designed pores; (ii) high-sensitivity optical and electrochemical sensing for real-time quality and safety signaling; and (iii) stimuli-responsive release of active agents (e.g., antimicrobials). We further explore the frontier of integrating HOFs as functional fillers or coatings within polymeric matrices, a key step toward practical devices. Despite challenges such as structural stability and maintaining permanent porosity due to relatively weak hydrogen bonds, this work aims to provide a design blueprint for advancing HOFs from laboratory curiosities to core components of sustainable, multifunctional packaging systems.

## 1. Introduction

The packaging industry is a core sector. It safeguards commodity circulation and enhances product value-added. Its development is deeply intertwined with food and drug safety, supply chain upgrades, and green environmental concepts. With the increasing consumer requirements for food and drug safety, quality traceability and environmental sustainability, conventional passive packaging fails to meet such evolving needs. Therefore, modern supply chains require new solutions: packaging functions are shifting from simple physical protection to intelligent dynamic regulation. As an innovative technology, smart packaging integrates sensing, response, and regulation capabilities. This allows it to perceive and respond to both internal and external environmental changes in real time, enabling dynamic product management [1].

Multifunctional porous materials (PMs) constitute a key research area in materials science and packaging engineering. Their importance continues to grow in diverse fields such as gas adsorption/storage, separation, catalysis, sensing, and environmental remediation [2]. Progress in this domain directly tackles major challenges within the global packaging sector, including the preservation of food and pharmaceuticals, monitoring quality and safety, and the development of sustainable packaging practices [2]. Owing to their unique pore structures and tunable functionality, PMs have undergone extensive preservation and environmental parameter monitoring. PMs can be categorized into inorganic [3] (e.g., zeolites), inorganic–organic hybrids [4] (e.g., metal–organic frameworks (MOFs)), and organic [5] (e.g., porous organic polymers (POPs)), with each type exhibiting distinct advantages and limitations in smart packaging applications. Conventional porous materials, such as zeolites, silica, and activated carbon, are commercially mature and benefit from low raw material costs. However, they all suffer from inherent limitations. Zeolites, for instance, as microporous crystalline aluminosilicates composed of AlO units, exhibit excellent thermal and chemical stability—properties useful for moisture control and odor removal in packaging [6,7]—their structural diversity is limited by a relatively monotonous set of building units. This limitation hinders their ability to meet the design requirements for advanced, smart responsive packaging systems. Meanwhile, mainstream crystalline porous materials also have significant shortcomings: Metal–organic frameworks (MOFs) are constructed from metal cores and organic ligands, forming one-, two-, or three-dimensional structures with high porosity and tunable functionality [8,9]. While MOFs demonstrate useful antimicrobial and gas-scavenging properties for food packaging [10], their practical application is hampered by moisture sensitivity, the potential risk of metal ion leaching, high production costs, and challenges in recycling [11]. Covalent organic frameworks (COFs) possess high crystallinity, structural flexibility, and a fully organic composition that avoids metal-related toxicity concerns [12,13,14,15]. Although they show excellent performance in gas separation and adsorption, their large-scale adoption in smart packaging is limited by demanding synthesis conditions and high manufacturing costs. Therefore, there is a surging demand for novel porous materials [9,12,16] to overcome bottlenecks like the inherent trade-off between adsorption capacity and selectivity [17], meeting the demands for high-quality, large-scale, and environmentally sustainable smart packaging.

Hydrogen-bonded organic frameworks (HOFs) are an emerging class of crystalline porous materials. Unlike MOFs and COFs, which are connected by covalent or coordination bonds, HOFs self-assemble through directional hydrogen bonds, supported by π–π stacking and van der Waals interactions [16]. The relatively weak bond energy and low directionality of hydrogen bonds allow a wide variety of HOF isomers to be obtained by varying solvents and reaction conditions, enriching their structural diversity. This flexibility (relatively weak bond energy) also enables HOFs to be readily synthesized under mild conditions using conventional crystallization methods, such as evaporation/cooling crystallization, or liquid–gas diffusion. Furthermore, the dynamic and reversible nature of hydrogen bonds grants HOFs good solubility in certain organic solvents. This characteristic not only improves their solution processability and recyclability but also provides potential for self-healing and structural reorganization [18]. Similar to MOFs and COFs, HOFs possess key porous material traits, including well-defined topology, high porosity, and large specific surface areas [19]. They further offer distinct advantages such as tunable pore sizes, modifiable pore surfaces, excellent solution processability, good recyclability and notable biocompatibility [20]. Compared to MOFs, HOFs lack metallic components, eliminating the risk of metal ion leaching. Their fully organic composition also contributes to lower density, reduced toxicity, and improved stability in humid environments [21]. Relative to COFs, HOFs are synthesized under milder conditions, are often recyclable, and offer considerable structural tunability. Through rational ligand design and hydrogen-bond engineering, diverse one-, two-, or three-dimensional frameworks can be constructed [22], allowing for precise control over pore size and architecture. Notably, HOFs incorporating polar functional groups can interact with specific gas molecules via hydrogen bonds, enabling selective adsorption and separation. This property has facilitated their widespread use in gas adsorption control and sensing [18,23], these combined advantages make HOFs ideal candidates for functional smart packaging, effectively overcoming key limitations of existing porous materials [24].

Despite significant progress, the relatively low bond strength of hydrogen bonds presents challenges for the development and application of HOFs. During activation, these bonds can be easily disrupted, leading to amorphization, structural collapse, or lattice rearrangement. This, in turn, hinders the establishment of clear structure-property relationships [25]. Compared to MOFs and COFs, HOFs generally exhibit lower structural stability and poorer thermal tolerance [26,27,28], and maintaining permanent porosity remains a persistent challenge in the field. These limitations have caused HOFs to lag behind the subsequently developed MOFs and COFs in both advancement and application. Nevertheless, recent studies have proposed effective molecular design strategies to enhance the stability of HOFs [29]. Furthermore, constructing HOF-based composites has also been demonstrated as a viable approach to extend their applications [26,30].

In our previous study [31], we established a foundational overview of the potential applications of Hydrogen-Bonded Organic Frameworks (HOFs) within the domain of smart packaging. This work significantly extends the discussion. We present a comprehensive and in-depth analysis that elucidates the specific functional mechanisms of HOFs—particularly their exceptional gas adsorption, controlled release, and sensing capabilities—which are crucial for intelligent packaging systems. By synthesizing the latest advancements in HOF-based applications, this review highlights their functional advantages while critically assessing current technological challenges and future trends. Ultimately, this analysis aims to provide a strategic framework to guide the rational design and practical implementation of HOFs in next-generation intelligent packaging.

## 2. Advantages of HOF Materials in Smart Packaging Applications

**Figure 1:**
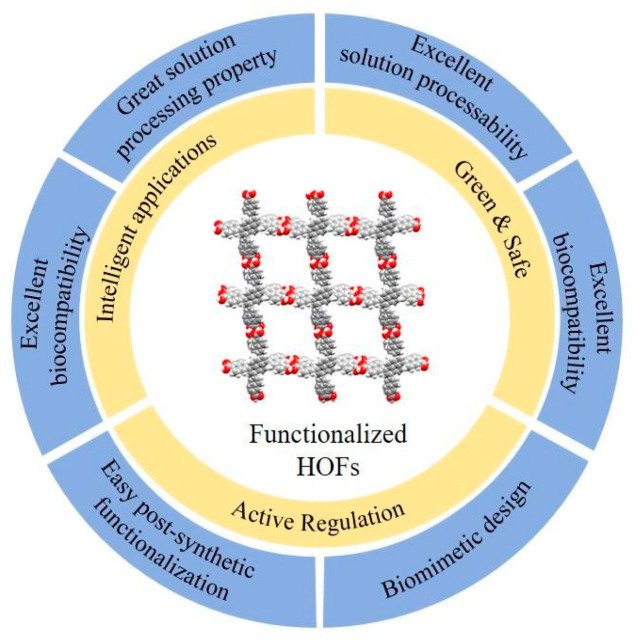
HOFs for Intelligent Packaging.

HOFs are crystalline materials assembled from organic building units through directional hydrogen bonds. Though weaker than the covalent bonds in COFs or the coordination bonds in MOFs, the hydrogen bonds in HOFs offer greater flexibility. This characteristic promotes structural diversity, even as it may somewhat limit mechanical robustness. As summarized in Figure 1, HOFs possess a combination of advantageous features, including structural design flexibility, tunable porosity, mild synthesis conditions, high crystallinity, solution processability, and ease of repair and regeneration. These attributes make HOFs promising candidates for functional smart packaging applications such as adsorption, separation, and sensing [19,24].

### 2.1. Designable Pore Structures for Precise Atmospheric Control

**Figure 2:**
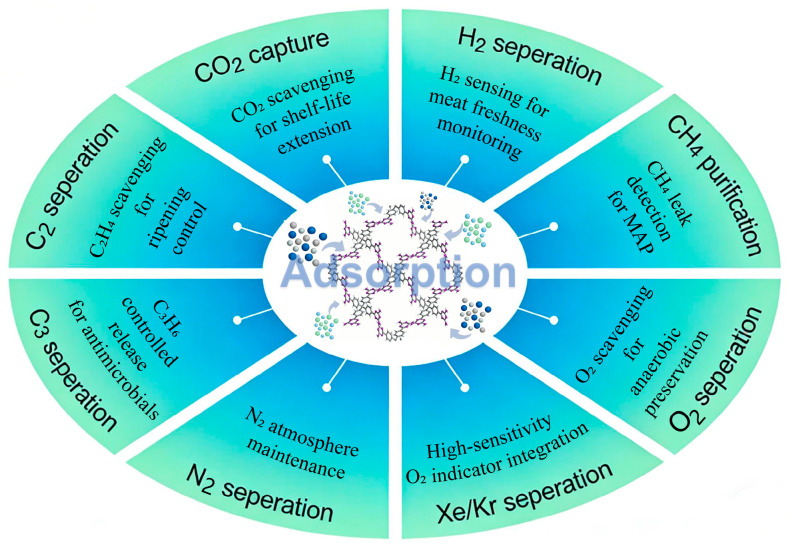
Multifunctional Applications of Hydrogen-Bonded Organic Framework Materials in Gas Capture and Separation.

Smart packaging requires functionality customized to the specific contents being protected. This may involve controlling the preservation atmosphere for fresh produce or dynamically monitoring the temperature and humidity of the product environment. Consequently, the design of packaging materials with targeted functions is a crucial step in developing smart packaging systems. Compared to traditional adsorbents such as activated carbon and zeolites, HOFs offer superior adsorption capacity and selectivity. They show significant potential in gas adsorption and storage (as shown in Figure 2), capable of effectively capturing various gases, including carbon dioxide, nitrogen, oxygen, ammonia, and hydrogen [32,33]. Their performance in gas separation is summarized in Table 1. Selectivity for specific gases can be further enhanced by optimizing the functional groups on the organic ligands [34]. Key factors that determine the topological structure and pore characteristics of HOFs include the type of ligand functional groups, the size of the molecular building blocks, and their geometric symmetry. By selecting appropriate molecular building blocks, HOF materials with tunable one-, two-, or three-dimensional topologies and adjustable pore sizes can be constructed. Ligand design plays a key role in this process. For instance, employing short-chain carboxyl-based ligands typically leads to microporous structures (pore size < 2 nm), as exemplified by HOF–16 and HOF–BTB. These materials often combine high stability with multifunctionality, allowing efficient adsorption and separation of small-gas molecules—a fundamental requirement for precise atmospheric control in smart packaging applications [35]. Recent research has further advanced the gas adsorption performance of HOFs. For instance, Wang et al. [36] developed a novel HOF material (ZJU-HOF-60a) by introducing electron-rich acetylene sites and controlling pore sizes, enabling preferential adsorption of ethane (reverse separation) to yield high-purity ethylene in a single step.

Hydrogen-bonded organic frameworks (HOFs) also show considerable potential for practical applications, including methane purification and CO_2_ capture. Zhou et al. [37] evaluated the performance of two three-dimensional HOFs, JLU-SOF2 and JLU-SOF3, for key methane purification scenarios. At 298 K and 1 bar, both materials exhibited excellent impurity removal. JLU-SOF2 showed separation selectivities of 16.3 and 48.1 for equimolar C_2_H_6_/CH_4_ and C_3_H_8_/CH_4_ mixtures, respectively. JLU-SOF3 achieved even higher values of 17.8 and 89.2 for the same gas pairs. Both frameworks maintained stable performance in separating CO_2_/CH_4_ mixtures, enabling efficient removal of CO_2_ impurities from methane streams. For CO_2_ capture, their CO_2_ adsorption capacities reached 1.98 and 2.34 mmol/g, respectively, under the same conditions. Moreover, their precisely tailored pore structure and strong host-guest interactions allow efficient separation of light hydrocarbons such as C_2_H_6_ and C_3_H_8_.

The hybrid matrix membrane based on HOF-21, developed by Wang et al. [38], exhibits a CO_2_ permeability of 840 Barrer and a CO_2_/N_2_ separation selectivity of 60 under humid conditions. This performance stems from its precise pore-size sieving effect, coupled with specific CO_2_ adsorption sites provided by Cu^2+^, bound water, and hydrogen bond networks, which work together to efficiently exclude N_2_. The material also demonstrates excellent stability in humid environments, a critical requirement for real-world packaging applications. This functionality makes it highly suitable for gas-flushing systems in smart packaging, where it can efficiently remove respiration-derived CO_2_ while maintaining a nitrogen-rich atmosphere that inhibits food oxidation and microbial growth. Furthermore, the inherent design flexibility and solution processability of HOFs allow them to be fabricated into films or coatings that can be integrated onto various packaging substrates, meeting the need for lightweight, functional packaging designs.

However, the relatively weak interactions in HOFs often challenge the stability of their framework structures, making the attainment of permanent porosity a key bottleneck for their use in gas adsorption and separation. This limited stability currently restricts the broader application of HOFs in these fields. Future research is likely to focus on designing and synthesizing novel organic building blocks to construct HOFs with robust, permanent porosity. A practical approach involves the rational design and assembly of ordered network frameworks, which could significantly enhance gas storage performance. Moreover, investigating the selective adsorption mechanisms between HOFs and specific gases has revealed that their building blocks can differentiate gas molecules through multiple weak interactions. This insight opens considerable potential for applications in gas adsorption and membrane-based separation, providing innovative pathways for the selective capture and separation of gases. Consequently, future efforts should prioritize the design of high-performance HOF materials tailored for gas separation. Engineering functional HOFs through precise pore-structure customization represents a promising development direction for gas separation membranes in smart packaging applications.

**Table 1:** Application of HOFs in Gas Separation.

	HOF Materials	Properties	Capacity/mmol/g	Qst/kJ mmol	Reference
CO_2_ capture	HOF-5a	CO_2_/CH_4_ separation	4.02 (296 K, 1 bar)	22.8	[39]
	HOF11a	CO_2_/CH_4_ separation	2.19 (273 K, 1 bar), 1.34 (296 K, 1 bar)	_	[40]
H_2_ Separation and Storage	ZJU-HOF-5a	H_2_ Storage	10.5 (77 K, 1 bar) (9.3 wt% at 100 bar)	5.2	[41]
	HOF-S	H_2_/CO_2_, H_2_/CH_4_ separation	0.268 (298 K, 1 bar)	7.38	[42]
	HOF-M	H_2_/CO_2_, H_2_/CH_4_ separation	0.446 (298 K, 1 bar)	6.40	[42]
	HOF-L	H_2_/CO_2_, H_2_/CH_4_ separation	0.625 (298 K, 1 bar)	6.18	[42]
CH_4_ purification	HOF16a	C_2_H_2_/CH_4_ purification	3.08 (273 K, 1 bar), 2.68 (298 K, 1 bar)	23.0	[43]
	JLUSOF3	C_3_H_8_/CH_4_ purification	5.51 (273 K, 1 bar), 4.70 (298 K, 1 bar)	42.7	[37]
	PFC-5	C_2_H_6_/CH_4_ purification	1.45 (273 K, 1 bar), 1.16 (298 K, 1 bar)	25.0	[44]
	PFC-2	C_2_H_4_/CH_4_ purification	2.75 (273 K, 1 bar), 1.83 (298 K, 1 bar)	23.7	[45]
O_2_ Separation	benzo-tris((trisfluoromethyl)-imidazole	O_2_/N_2_ separation	50 cm^3^/g (77 K, P/P_0_ = 0.90)	_	[46]
Xe/Kr Separation	HOF-40	Xe/Kr separation	1.56	_	[47]
	HIAM-103	Xe/Kr separation	1.39 (298 K, 1 bar)	24.5	[48]
N_2_ separation	JLU-SOF2	CO_2_/N_2_ separation	4.25(273 K, 1 bar), 1.98 (298 K, 1 bar)	24.5	[37]
	JLUSOF1-R	CO_2_/N_2_ separation	3.39 (273 K, 1 bar), 2.37 (298 K, 1 bar)	34.3	[49]
	CPOS-5	CO_2_/N_2_ separation	2.14 (273 K, 1 bar)	34.5	[50]
C_3_ Separation	HOFFJU-1a	C_3_H_6_/C_3_H_8_ separation	2.2 (298 K, 1 bar)	32.4	[51]
	HOF-30a	C_3_H_4_/C_3_H_6_ separation	2.67 (298 K, 1 bar)	40.3	[52]
C_2_ Separation	HOF-FJU-1a	C_2_H_4_/C_2_H_6_ Separation	2.10 (298 K, 1 bar)	_	[53]
	HOF-3	C_2_H_2_/CO_2_	2.59 (273 K, 1 bar), 2.10 (296 K, 1 bar)	<20	[54]

### 2.2. Superior Solution Processability for Facile Manufacturing

The industrial adoption of smart packaging depends heavily on whether functional materials can be integrated with established packaging processes—such as film formation and coating—while maintaining cost competitiveness. For hydrogen-bonded organic frameworks (HOFs), the reversible nature of their intermolecular interactions grants them excellent solution dispersibility. Many HOFs readily disperse in mild, green solvents such as water or ethanol, forming stable colloids or suspensions. This process typically avoids harsh conditions like high temperatures, high pressures, or corrosive reagents, making it environmentally benign and helping to preserve the material’s structure and properties. As a result, HOFs can be processed into films using common, low-cost techniques like solution casting, spin coating, and spraying. Their favorable solution processability highlights their strong potential for smart packaging applications [55]. The solution processability also facilitates material restructuring. The reversible hydrogen bonds allow broken networks to reconnect in solution, enabling healing and regeneration of the original HOF structure. Such capability further supports the shaping and pelletization of bulk HOF materials [56]. Moreover, the ease of introducing substituents onto the organic building blocks allows straightforward functionalization of HOFs, which synergizes effectively with their solution processability. This characteristic enables the preparation of HOFs–polymer hybrid matrix membranes, which enhance the performance of the film and overcome the limitations of pure HOFs or pure polymer. For example, Wang et al. [57] prepared hybrid matrix films by incorporating HOF-101 crystals into a polyvinylidene fluoride (PVDF) matrix. The enhanced molecular chain interactions significantly improved the film’s mechanical strength and impact resistance, while also imparting low-temperature antiviral activity—a promising example for multifunctional smart packaging. Additionally, the solution processability of HOFs makes them compatible with common packaging production lines, such as cast film formation, spraying, and electrospinning. Yang et al. [58], for instance, successfully combined HOF materials (MA-IPA@NPA) with polyvinyl alcohol (PVA) nanofibers via electrospinning technology. The resulting composite acts as a fluorescent sensor for detecting tryptamine and gallic acid in food, addressing safety and food security concerns.

When comparing the solution processability of HOFs with that of conventional packaging polymers (Polyethylene (PE), Polypropylene (PP), Polyethylene terephthalate (PET), Polylactic acid (PLA))—HOFs demonstrate notable strengths in mild processing conditions, functional tunability, and greener production. These advantages align well with the growing demand for low-carbon and high-value-added smart packaging. Widely used polymers like PE and PP can be processed into films via melt extrusion at mild temperatures around 150–200 °C, enabling relatively low-energy fabrication. However, such polyolefins are structurally inert and lack responsive, adsorptive, or sensing functionalities required for intelligent packaging. PET, on the other hand, has a much higher melting point (250–280 °C) and requires harsher thermal conditions along with high-temperature melt stretching, resulting in significantly higher energy consumption and a larger carbon footprint. Even PLA, a representative biodegradable polymer with relatively moderate processing requirements, still depends on melt processing at 160–220 °C and offers limited functional versatility. In contrast, HOFs can be processed at low-temperature through dispersion in green solvents. This approach reduces energy consumption and avoids thermal degradation of functional components. HOFs further combine solution processability with structural functionalization, enabling the one-step preparation of multifunctional materials with sensing, antibacterial, or gas separation properties [59]—capabilities that traditional polymers typically require complex post-modification processes to achieve. Meanwhile, most current research on HOF-polymer composites has focused on niche polymers such as PVDF and PVA [57,59,60,61]. Further exploration is needed regarding the compatibility of HOFs with commodity packaging polymers like PE, PP, and PET, which dominate the global packaging market. The polar characteristics of most HOF particles may lead to interfacial incompatibility with non-polar polymers such as PE and PP, potentially affecting dispersion uniformity and film-forming performance. Targeted strategies, such as surface modification of HOFs, will be key to improving this matching degree for broader industrial application.

As research on the solution processability of HOFs advances, it is equally important to broaden the evaluation scope to encompass high-throughput industrial processing routes, food contact safety and full life cycle environmental impact. While HOFs have been effectively processed at the lab scale via casting, spraying and electrospinning [58,59,62], extending these processing advantages to mainstream industrial packaging techniques such as melt extrusion, blown film formation and continuous roll-to-roll coating will be the next key research focus. For melt extrusion, the dominant industrial route for producing PE and PP packaging films, systematic assessments of the thermal stability of HOFs under high-temperature extrusion conditions is essential. This will help preserve the crystalline framework integrity and intrinsic functional performance of HOFs during large-scale production. For food contact smart packaging, a pivotal application scenario for HOF-based materials, systematic datasets regarding the migration of HOF fragments or organic building blocks into food simulants under typical storage conditions (fluctuating temperatures, variable pH values, and extended contact times) remain to be established; additionally, compliance with international food contact regulations (e.g., Commission Regulation (EU) No 10/2011 (EU 10/2011) and Title 21 of the Code of Federal Regulations (FDA 21 CFR)) is a prerequisite for successful commercial translation. In contrast, conventional packaging polymers (PE, PP, PET, and PLA) possess well-defined migration limits and full regulatory approval for direct food contact, offering a definitive benchmark for the safety evaluation and regulatory compliance of HOF-based materials. Beyond manufacturing, HOFs already offer environmental benefits through their green low-temperature, solution-based processing, which effectively reduces carbon emissions during production.

### 2.3. High Surface Area for Multi-Functional Loading and Synergy

Hydrogen-bonded organic frameworks (HOFs) offer advantages for smart packaging that extend beyond their tunable pore architecture. The synergistic between their high specific surface area and open-channel structure supports multiple concurrent adsorption mechanisms—including hydrogen bonding, electrostatic interactions, π-π stacking, and Lewis acid-base coordination—particularly in the presence of macromolecules or highly charged ions. This further enhances the materials’ adsorption performance and functional expansion capabilities [56]. Compared with traditional functional packaging materials, the high specific surface area of HOFs maximizes the exposure of active adsorption sites, thereby improving their effectiveness. Mechanistically, this large surface area increases the frequency of contact between the material and target gases, boosting adsorption efficiency. More importantly, it enables higher loadings of functional agents (e.g., fluorescent probes, antimicrobial agents), facilitating the development of multifunctional smart packaging.

Numerous studies have successfully developed multifunctional materials by leveraging the synergistic properties of HOFs. For instance, Wang et al. [63] created a photosensitive HOF-based coating that converts adsorbed oxygen into reactive oxygen species (ROS) under natural sunlight and stores them, thereby imparting long-lasting self-cleaning and antibacterial capabilities. Zhang et al. [64] prepared crystalline HOF-16 membranes via a methanol vapor-induced in situ conversion strategy. The membranes exhibit a BET surface area of 185.1 m^2^/g and a well-ordered porous channel structure. The high surface area provides abundant pathways for gas transport while fully exposing the functional groups within the pores, significantly enhancing hydrogen permeation flux and separation selectivity. Other work has further extended the synergy between high surface area and pore design. For instance, ionic HOFs such as PFC–33 have been constructed using porphyrin photosensitizers as the framework, with antimicrobial agents incorporated as counterions [65]. This design achieves ion-responsive release behavior, demonstrating synergistic photodynamic and chemical antibacterial effects. The ability to respond to environmental stimuli (such as light, ion concentration, temperature, and humidity) and release active substances makes it suitable for the “smart release” requirements in smart packaging. Thus, the combined designability of pore structures and high specific surface area in HOFs not only enhances adsorption and loading capabilities but also enables responsiveness to external stimuli and multifunctional integration. This provides critical support for evolving smart packaging from single-function systems toward multifunctional, intelligently responsive platforms.

### 2.4. Biocompatibility and Environmental Friendliness for Green Development

In an era of increasing environmental awareness and emphasis on green safety, the development of eco-friendly, bio-based packaging materials that combine safety with functional has become a key trend. Traditional packaging materials typically consist of polymers, most of which are difficult to degrade. Owing to their distinctive porous architecture and high specific surface area, metal–organic frameworks (MOFs) show considerable potential for active and intelligent food packaging. However, their use in smart packaging involves certain drawbacks. Functional materials containing metal components may carry risks of metal ion leaching, which can compromise product safety [66]. Zhao et al. [67] systematically investigated the toxicological mechanisms and safety risks of MOFs in food packaging, confirming that their toxicity mainly stems from the leaching of metal ions, dissociation of organic ligands, excessive release of loaded bioactive agents, and residual solvents from synthesis. Uncontrolled release of metal ions (e.g., Cu^2+^, Ag^+^, Co^2+^) can lead to migration into food, causing contact-induced cellular damage and accumulation in organs. Notably, the “Trojan Horse” effect of intracellular toxicity remains a critical concern in the food-contact scenarios of smart packaging, and conventional detection methods often fail to accurately assess the hazards associated with their nanoscale migration.

In contrast, metal-free HOFs avoid the core metal-related toxicity concerns of MOFs. Owing to their purely organic, hydrogen-bonded architecture, they exhibit superior biocompatibility and lower toxic potential, better aligning with the direct food-contact safety requirements of intelligent packaging. HOFs are assembled solely through non-covalent interactions such as hydrogen bonds and π-π stacking, containing no metal ions. This eliminates the risk of releasing harmful metal pollutants during degradation and avoids associated toxicity issues [68]. The reversible nature of hydrogen bonds in HOFs facilitates easier dissociation and reassembly, further enhancing their biocompatibility. As a result, HOF-based environmentally friendly multifunctional porous materials and drug-delivery systems hold considerable promise [69]. Beyond their inherent safety, the building blocks of newer bio-based HOFs are often derived from biocompatible small organic molecules or their derivatives (such as amino acids). Upon degradation, their breakdown products are generally small, benign molecules such as water and carbon dioxide, in line with the principles of green chemistry and sustainable development [70]. This provides important toxicological evidence supporting HOFs as a promising alternative to MOFs for intelligent food-packaging applications [71]. Building on these foundations, HOFs demonstrate practical application value in multiple aspects of smart packaging. They show unique advantages in detecting food contaminants [72,73,74] and enable selective adsorption within complex food matrices [75], paving the way for highly effective preservatives to extend shelf life. Simultaneously, HOFs can serve as functional additives in food packaging materials, enhancing safety and functionality through inherent antimicrobial and antioxidant properties. Furthermore, their inherent safety and non-toxicity allow HOFs to be used in developing novel sensors for rapid, sensitive detection of harmful substances in food [26,76], offering strong support for food safety regulation.

In practical applications, HOF materials have achieved multiple validated successes in the field of safe, bio-based packaging. For example, Tang et al. [77] developed an innovative strategy for the in situ encapsulation of proteins within nanoscale HOFs. This mild assembly process preserves the native spatial structure of proteins, thereby maintaining the high biological activity of enzymes and other biomolecules—a key advantage for loading active substances into packaging. In another study, Ke et al. [78] explored the use of pyridyl-functionalized HOFs in ethylcellulose (EC)-based food packaging films to develop eco-friendly packaging materials. They synthesized two types of HOFs, HOF-PyTTA and PFC-2, and prepared EC-HOF composite films via solution casting. The resulting films not only showed excellent biocompatibility and hemocompatibility but also exhibited significantly enhanced UV-blocking and antioxidant properties. Moreover, the incorporation of HOFs substantially improved the mechanical properties and thermal stability of EC films, offering new insights for the green design of smart packaging materials.

## 3. Application Mechanisms and Development Trends of HOF Materials in Smart Packaging

### 3.1. Gas Adsorption and Response Mechanisms of HOF Materials in Smart Packaging

In recent years, regulating the internal gas environment to inhibit microbial growth and food oxidation has been a core requirement for smart packaging materials. Excessively high CO_2_ concentrations can lead to food browning and texture softening. Nitrogen (N_2_), as an inert gas, can effectively exclude oxygen to prevent oxidation while also providing cushioning protection for the packaged product. HOF materials show exceptional practical value in this regard due to their highly selective adsorption of CO_2_/N_2_. The framework of HOFs exhibits strong affinity for CO_2_, whereas interactions with N_2_ are generally weak. As a result, most HOFs display outstanding CO_2_/N_2_ selectivity [44]. Such materials can establish a high-N_2_, low-CO_2_ atmosphere that suppresses microbial growth and oxidative deterioration, effectively extending food shelf life—a key advantage for smart packaging applications.

Notably, unlike traditional adsorbents such as activated carbon and zeolites, HOF materials can achieve selective adsorption of specific gases by optimizing functional groups on organic ligands (e.g., introducing amino, carboxyl, or hydroxyl groups) [79]. Gas adsorption in HOFs primarily relies on two mechanisms: pore size exclusion and enhanced hydrogen-bond interactions. When the pore size of HOF matches the kinetic diameter of a target gas molecule, size-based selectivity can be achieved. Additionally, hydrogen-bond donors present in the HOF ligands can form favorable interactions with certain gas molecules, further strengthening adsorption [80]. This hydrogen-bond enhancement is facilitated by the donor sites within the framework. For instance, Wang et al. [42] synthesized a carboxyl-functionalized HOF membrane (HOF-S) with a pore size of 0.62 nm. This membrane synergistically utilizes size-exclusion effects and electrostatic interactions generated by abundant carboxyl groups within the pores to effectively impede CO_2_ diffusion, thereby enhancing selectivity toward H_2_/CO_2_.

Additionally, membrane-based gas separation offers advantages over other separation techniques, including low energy consumption, simple operation, and a small environmental footprint. While porous materials like zeolites, metal–organic frameworks (MOFs), and covalent organic frameworks (COFs) have been successfully used to fabricate gas-separation membranes, studies on hydrogen-bonded organic frameworks (HOFs)—which benefit from mild synthesis and straightforward preparation—remain limited. Recently, Feng et al. [56] reported a microporous HOF-based separation membrane (UPC-HOF-6) constructed from 4′, 4″, 4‴-nitrilotris (([1,1′-biphenyl]-4- diaminotriazine)) (NBP-DAT). Rich in hydrogen bonds and strong π-π interactions, the UPC-HOF-6 membrane exhibits excellent H_2_/N_2_ separation performance. Applying this principle to smart packaging allows the design of HOF materials that can selectively remove specific gases while preserving beneficial ones, thereby maintaining an optimal internal atmosphere with precision.

In addition to their excellent gas adsorption and separation performance, the tunable pore structure of HOFs allows the loading of active substances, enabling responsiveness to external environments. This provides a theoretical foundation for dynamic responses in smart packaging. Research on the responsive functions of HOFs has progressed significantly in recent years, allowing real-time monitoring and response to various substances, including gases. For example, He et al. [81] developed a novel semiconductor gas sensor using a sulfonic acid-based HOF. Leveraging the material’s ordered porous structure and p-type semiconductor properties, they achieved effective ammonia sensing. The sensor showed superior response/recovery times (18 s/15 s at 0.005 mL/L), high sensitivity, excellent reproducibility, and stability, offering new insights for gas sensing applications of HOFs. Liu et al. [65] investigated ionic HOFs, in which permanent channels and electrostatic interactions between the framework and counterions confer ionic responsiveness. This enables the controlled release of agents such as biocides in specific physiological environments. Such work provides a foundation for designing smart packaging that can respond to specific ions or metabolites generated during food spoilage.

### 3.2. Sensing and Monitoring Mechanisms of HOF Materials in Smart Packaging

Smart packaging requires real-time monitoring of storage conditions (humidity, temperature) and the status of both packaging and contents (active ingredient degradation, seal integrity). Sensor systems built from HOF materials, which leverage their optical and electrical properties, enable highly sensitive detection of these critical indicators. Due to their porosity, tunable ligand types, and diverse functional sites, HOF materials show considerable promise for sensing applications. The microenvironment inside their pores can recognize and concentrate specific guest molecules. Interactions between these guest molecules and the HOF framework can alter the fluorescent properties of the material, allowing HOFs to be used in fluorescence-based sensing [16,82]. Representative applications of HOF-based sensing in food-safety analysis are summarized in Table 2.

By introducing polar functional groups (e.g., carboxylic acids, amino groups) or by functionalizing with lanthanide ions, HOFs can be endowed with outstanding optical properties and sensing capabilities. Moreover, the relatively low energy of hydrogen bonds allows precise control over ligand concentration in solution, facilitating the formation of diverse HOF architectures. This structural variety is advantageous for designing hydrogen-bonded frameworks with photo-or electro-responsive characteristics [83]. Li et al. [16] developed HOFs through intermolecular hydrogen bonding interactions. These served as the structural foundation for a pH-sensitive fluorescein (5-FITC) conjugate-loaded and lanthanide Eu^3+^ coordination-doped HOF-FITC/Eu fluorescent probe, enabling real-time, intuitive detection of seafood freshness. Zhang et al. [84] prepared iron-modified hydrogen-bonded organic frameworks (Fe–HOFs) as fluorescent sensors for rapid ascorbic acid detection. Ke et al. [85] synthesized a novel HOF (FJU-200) via solvothermal synthesis using N,N-bis(5-isophthalic acid)naphthalenedicarboximide (H4L) as the starting material. This material detects aniline and UV light through dual signals of visual color change and fluorescence sensing. Results demonstrated that FJU–200 enables UV detection within minutes. The sensing and detection capabilities of HOFs provide novel insights for the functional design of smart packaging.

The persistent issue of counterfeit and inferior goods significantly undermines consumer rights. Conventional anti-counterfeiting technologies are often limited by their ease of replication, driving the need for new materials with unique structures and properties. Beyond sensing, hydrogen-bonded organic framework (HOFs) also show excellent potential for smart anti-counterfeiting packaging. Yang et al. [58] designed and synthesized a novel hydrogen-bonded organic framework material, MA-IPA@NPA, for application in smart anti-counterfeiting and food safety detection. This material exhibits unique dual fluorescence-phosphorescence emission with a long phosphorescence lifetime, offering both outstanding anti-counterfeiting performance and fluorescence sensing capability for tryptophan and sulfite. By electrospinning this material with polyvinyl alcohol (PVA), the researchers prepared composite nanofiber membranes to construct multi-response anti-counterfeiting labels. When combined with machine-learning-assisted multi-level verification, this approach holds promise for smart anti-counterfeiting and secure packaging. Yang et al. [86] designed a Eu^3+^-functionalized HOF(Eu@GC_2_-2) that acts as a paper-based sensor with high selectivity and detection efficiency for the pesticides thiram and caffeic acid. The team further developed a series of anti-counterfeiting inks based on Ln^3+^-functionalized HOFs, integrating neural networks and code conversion to create a multi-stimulus-responsive luminescent anti-counterfeiting platform. This system enables multi-layer information encryption and rapid identification, significantly increasing the difficulty of counterfeiting, and holds broad application prospects in the field of smart packaging anti-counterfeiting.

**Table 2:** Application of HOFs-based sensing methods in food safety analysis.

Materials	Sensor Type	Linear Range	Recovery	LOD	Sample	Reference
Tr-HOF	Electrochemiluminescence	1 nM–10 μM	83.0–105.0%	0.28 nM	Milk	[74]
Eu@Tt-TPA	HPLC-DAD	10^−8^–10^−4^ M	96–106%	0.87 ppm	Tap water	[87]
Lumi-HOF@Tb	Chemiluminescence sensor	0.1–30, 0.01–5 U/L	93.0–106.4%	0.04, 0.005 U/L	α-Glucosidase	[88]
HOF-FAFU-1	Fluorescent sensor	0–0.45 mM	99.1–100.1%	1.32 μM	Tap water	[89]
Ln@MA-DPA	luminescent sensor	0–30 μM	_	0.72 μM	Water	[76]
Fe-HOF	Fluorescent sensor	0.5–8 μM	_	0.14 μM	Vitamin C pills, beverages	[84]

### 3.3. Integration and Interface Functionalization Mechanisms of HOF Materials in Smart Packaging

Hydrogen-bonded organic frameworks (HOFs) show significant promise for smart packaging applications. However, constructing HOF/polymer hybrid films that simultaneously maintain structural and functional integrity remains challenging. Against this backdrop, integrating HOFs into polymer matrices to build HOF/polymer hybrid films represents a viable approach, validated by its feasibility in manufacturing MOF/polymer hybrid film materials [90]. To translate HOFs into industrially practical packaging films or functional coatings, several critical issues must be addressed. These include processability, dispersibility, structural stability, and functional synergy. This necessitates resolving compatibility and interfacial bonding stability with common packaging substrates such as polyethylene, polylactic acid, and polyvinylidene fluoride. Currently, the integration of HOFs with polymer substrates is primarily achieved through three methods: covalent bonding, physical blending, and heterogeneous nucleation-guided growth. The distinct interfacial mechanisms directly influence the structural integrity and smart response efficiency of the composite materials.

**Figure 3:**
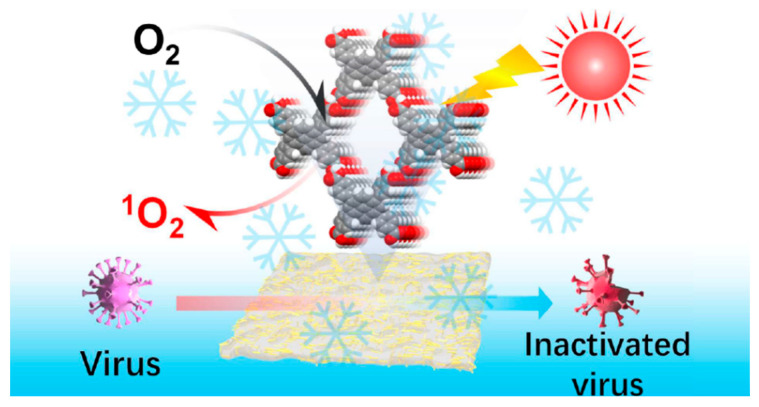
Schematic Diagram of Cold Chain Smart Antiviral Packaging Application Based on HOF-101@PVDF Composite Membrane [57].

Physical blending is currently the simplest and most industrially compatible method for integrating HOFs with packaging substrates. By incorporating HOF nanocrystals as functional fillers into flexible substrates such as polyvinylidene fluoride (PVDF) and polylactic acid (PLA) via methods like solution casting and electrospinning, materials can be endowed with sensing, adsorption, and antimicrobial functions without significantly altering existing packaging processing techniques. During this process, interfacial interactions between HOFs and polymers—such as hydrogen bonding and van der Waals forces—improve particle dispersion, reduce interface defects, and achieve synergistic enhancement of mechanical properties. However, attention must be paid to the leakage of HOF particles within the polymer matrix [91]. Wang et al. [57] used solution casting to combine HOF-101 crystals with PVDF, producing an HOF-101@PVDF hybrid matrix film. Hydrogen bonding between HOF-101 and PVDF strengthened the interface, significantly improving the mechanical performance of the composite. It demonstrated excellent antiviral performance at low temperatures while maintaining good cycling stability, offering a new green and long-lasting solution for antiviral protection in low-temperature food packaging (Figure 3).

Introducing reactive functional groups into HOF ligands structure to form covalent crosslinks with polymer segments is an effective approach to enhance framework dispersion and structural stability. Covalent bonding offers greater stability. By utilizing in situ polymerization to chemically anchor HOFs within the polymer network, interfacial gaps and uneven distribution of HOFs within the polymer matrix can be effectively avoided [92], providing a viable approach for fabricating stable functional membranes resistant to solvents, acids, and alkalis. Related studies [93] reported a polymerizable HOF (HOF-50) constructed through charge-assisted hydrogen bonding and functionalized with allyl groups. This HOF was co-polymerized in situ with butyl methacrylate (BMA) and acrylamide (AAm) via photo-initiated polymerization. The resulting covalently bonded HOF/polymer hybrid films (polyHOFs) retain framework crystallinity and pore structure while exhibiting superior mechanical properties and chemical stability. This covalent approach addresses common issues such as HOF leaching and poor interfacial compatibility encountered in conventional physically blended composites.

To further enhance the compactness, separation, and barrier properties of composite membranes, researchers have adopted a strategy of heterogeneous nucleation and directed growth. Using nanomaterials such as MOFs as templates, they induce the in situ crystallization of HOFs on the template surfaces, resulting in hierarchical porous composite structures. These core–shell or interpenetrating architectures integrate the advantages of both porous materials, simultaneously enhancing gas permeability, selectivity, mechanical stability, and fatigue resistance. The HOF composite membrane grown from MOF nanosheets [94] exhibits significantly improved hydrogen permeability and separation selectivity compared to pure HOF membranes. It demonstrates excellent pressure response characteristics and long-term operational stability, providing an efficient strategy for fabricating high-performance gas separation membranes.

The integration of hydrogen-bonded organic frameworks (HOFs) with packaging substrates, along with the functionalization of their interfaces, is crucial for translating laboratory materials into industrial applications. Through precise interface design and structural regulation, future developments may yield HOF-based smart packaging materials that combine high stability, high responsiveness, and environmental compatibility. Such materials could integrate multiple functions—including anti-counterfeiting labels, atmosphere control, and freshness monitoring—further expanding the application scope of hydrogen-bonded organic frameworks in high-end smart packaging.

## 4. Issues and Development Trends of HOF Materials in Smart Packaging Applications

With the continuous advancement of smart packaging technology, packaging is increasingly becoming an intelligent component for product quality monitoring. Conventional smart packaging materials are often limited by their single functionality, failing to meet multifunctional requirements. Consequently, the development of multifunctional, biocompatible packaging materials has emerged as a key trend in the packaging industry [95]. HOF materials show considerable promise for smart packaging due to their tunable pore structures, high specific area, biocompatibility, and adsorption/detection capabilities. The tunable pores allow selective gas adsorption; the high specific surface area offers ample active sites for adsorption and detection, enhancing efficiency; and excellent biocompatibility avoids the risks associated with traditional metal-based functional materials [96,97]. The distinctive properties of HOF materials confer unique advantages but also present challenges for application. (1) Although the flexible, designable frameworks of HOFs enable adaptation to various processing needs, this flexibility can come at the expense of structural rigidity and pore stability. In practical smart packaging applications, external fluctuations may induce framework deformation or even pore collapse, degrading performance. Therefore, balancing flexibility and stability becomes a critical design factor. This may require reinforcing the framework through additional interactions like π-π and C–H-π stacking to ensure reliable performance in complex environments [24]. (2) The inherent properties of hydrogen bonds also pose challenges for HOFs applications. Compared to the strong, directionally defined coordination bonds in metal–organic frameworks (MOFs), the weak and non-directional nature of hydrogen bonds complicates the precise synthesis of HOFs structures. Extending building units or introducing functional groups readily alters the final material structure, while varying synthesis conditions yield different outcomes [19,98]. Therefore, overcoming the limitations of hydrogen bonding to achieve precise, reproducible HOF synthesis remains a critical direction for future advancement.

**Figure 4:**
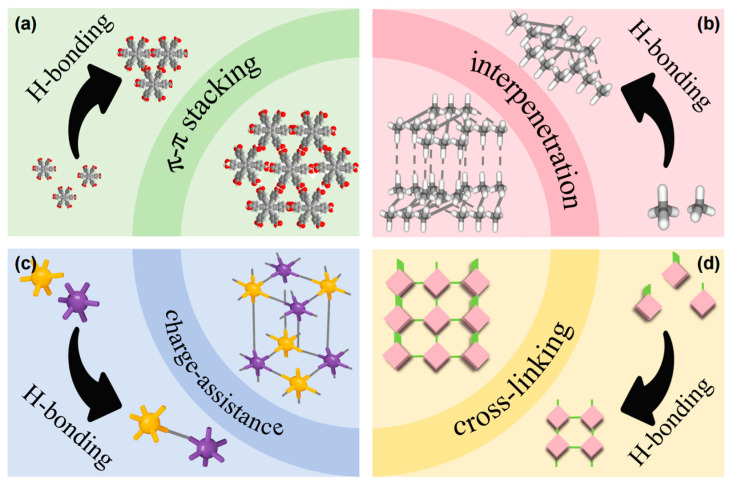
Representative strategies for the preparation HOFs with enhanced stability: (**a**) π-π stacking, (**b**) interpenetration, (**c**) charge-assisted Hbonding, and (**d**) cross-linking modification.

Currently, strategies such as employing rigid linkers [18] and regulating interpenetrating framework structures [99,100] have been proven effective in enhancing the structural stability of hydrogen-bonded organic frameworks (HOFs). Additionally, rigid scaffolds capable of forming multiple hydrogen bonds through specific interaction modes have demonstrated strong forces and directionality, proving effective in constructing HOFs with targeted structures. Theoretical calculations have even been successfully applied to predict and design the structures and properties of HOFs [101,102,103]. However, these strategies alone are insufficient for designing highly stable HOFs, as relying solely on hydrogen bonding makes it challenging to achieve excellent thermal and chemical stability. Consequently, researchers are focusing on developing design strategies for HOFs with accessible permanent porosity and high stability. Beyond utilizing complementary hydrogen bonds and multi-bonding sites as building blocks, key strategies to enhance HOFs stability include incorporating π-π stacking interactions, framework interpenetration, introducing charge-assisted hydrogen bonds, and chemical crosslinking modifications (Figure 4). Some HOFs with ultra-high stability (maintaining crystallinity under strong acids, strong bases, and boiling water) and ultra-large specific surface areas (maximum specific surface area up to 3400 m^2^/g) have also been synthesized [101]. Additionally, the absence of metal ions in HOF materials endows them with excellent biocompatibility and safety, garnering significant attention in fields such as bio-coatings, antimicrobial applications, and bio-detection [104]. In the field of smart packaging, the biocompatibility of HOF materials enables their application in food contact and pharmaceutical packaging. Further development of HOF materials can broaden their application scope. By precisely designing the properties of HOF materials for different application scenarios, they can be utilized in various applications such as gas separation, catalysis, sensing, biomedical, and antimicrobial applications [105]. While the unique properties of HOFs have attracted considerable interest, challenges remain, including insufficient stability of their structure and porosity, as well as difficulties in achieving multifunctionality [106]. Therefore, developing stable, multifunctional HOFs is of great significance for advancing smart packaging.

Despite the promising advantages of HOFs for food and packaging applications, several practical limitations and challenges to industrial scalability remain. While biocompatible and environmentally friendly HOFs (such as those constructed from amino acid derivatives and small organic molecules) have been developed [107,108,109], most reported HOFs still rely on structurally complex organic ligands that require multistep synthesis. This leads to high cost, low yield, and difficulties in large-scale production. Additionally, the structural stability of HOFs under conditions of high humidity, mechanical shear, and long-term storage needs further improvement to meet demanding industrial environments. Furthermore, poor compatibility between polar HOF particles and non-polar commodity polymers (PE, PP, PET), along with insufficient studies on food-contact safety and regulatory compliance, also hinders their practical adoption. These bottlenecks must be addressed before HOFs can be widely applied in industrial food packaging and safety monitoring.

Looking ahead, the advancement of hydrogen-bonded organic framework materials (HOFs) in food safety will achieve systematic breakthroughs through material design, functional expansion, and engineered applications. Future research will focus on precisely tuning the intrinsic properties of HOFs. This involves enhancing intermolecular interactions, optimizing structural stability, and adopting safer organic structural units suitable for food applications. Concurrently, efforts will improve structural stability and biocompatibility in complex food environments. Leveraging tunable pore structures will enable functional optimization for controlled release of active substances, targeted adsorption of contaminants, and efficient recognition. In sensing and detection, highly selective and sensitive HOF-based sensing systems will be developed to enable the transition from laboratory analysis to on-site, portable, and real-time food safety monitoring. In food storage and smart packaging, research will evolve from single-function exploration toward multifunctional HOF-based composites that integrate preservation, adsorption, and antimicrobial properties. Interface engineering will optimize compatibility with packaging substrates, facilitating efficient integration and controlled preparation of functional materials within packaging systems. Meanwhile, to meet the requirements of green and sustainable development, environmentally friendly preparation routes such as aqueous phase and mechanochemical synthesis will be prioritized. This trend will promote the translation of HOFs from lab-scale synthesis to low-energy, low-residue, and scalable industrial production. Furthermore, aligning with next-generation smart packaging trends, research will center on developing self-healing mechanisms, dynamic response systems, and intelligent monitoring capabilities based on HOFs. This will enable real-time food quality monitoring, precise shelf-life regulation, and comprehensive safety assurance throughout the packaging lifecycle. The paradigm shift toward intelligent packaging, which requires active interaction with products and the surrounding environment, has fundamentally challenged the passive characteristics of traditional packaging materials.

## 5. Conclusions

The paradigm shift toward intelligent packaging, which demands active interaction with the content and environment, has fundamentally challenged the passive nature of traditional packaging materials. This transition has catalyzed the search for advanced materials capable of multifunctionality. Hydrogen-bonded organic frameworks (HOFs), with their unique synergy of structural precision, biocompatibility and processing versatility, have emerged as a particularly promising candidate to meet these complex requirements.

By outlining the structure and properties of HOFs, this study highlights their distinct advantages for smart packaging applications. Specifically, HOFs exhibit exceptional performance in three core functional domains: selective gas adsorption, responsive molecular release, and sensitive target detection—collectively representing the foundational pillars of intelligent packaging systems. On the basis of their applications as gas adsorbents for controlled atmosphere storage, sensory probes for real-time food quality monitoring, and functional fillers for antimicrobial packaging, HOFs are expected to drive the gradual transition of food preservation from traditional passive protection to modern intelligent management. Despite these promising advances, critical bottlenecks still hinder their industrial translation. Specifically, structural design must balance flexibility and stability to ensure efficient adsorption and structural integrity, while the relatively weak hydrogen-bonding interactions within HOFs complicate their precise structural synthesis.

Looking forward, HOF-based smart packaging is poised to evolve from single-function demonstrations to integrated, sustainable solutions. Rational molecular design can be employed to enhance framework stability, optimize pore architectures for targeted guest recognition, and promote scalable manufacturing. These strategies will facilitate the development of next-generation packaging systems that actively preserve food quality, dynamically monitor safety, and minimize environmental impact. Addressing the aforementioned inherent challenges is critical to unlocking the full potential of HOFs for innovative smart packaging solutions. HOFs are poised to serve as next-generation, green, and high-efficiency functional materials, driving continued innovation and sustainable development in smart packaging.

## Data Availability

No new data were created or analyzed in this study. Data sharing is not applicable to this article.
